# Risk of Idiopathic Dilated Cardiomyopathy in 29 000 Patients With Celiac Disease

**DOI:** 10.1161/JAHA.112.001594

**Published:** 2012-06-22

**Authors:** Louise Emilsson, Bert Andersson, Peter Elfström, Peter H.R. Green, Jonas F. Ludvigsson

**Affiliations:** Vårdcentralen Värmlands Nysäter, Värmland County, and the Department of Medicine, Örebro University, Örebro, Sweden (L.E.); Institute of Medicine, Department of Molecular and Clinical Medicine, Sahlgrenska Academy, University of Gothenburg, Gothenburg, Sweden (B.A.); Department of Neonatology, Astrid Lindgren Children's Hospital–Danderyd, Karolinska University Hospital, Stockholm, Sweden (P.E.); Department of Medicine, Columbia University College of Physicians and Surgeons, New York, NY (P.H.R.G.); Department of Pediatrics, Örebro University Hospital, and the Clinical Epidemiology Unit, Department of Medicine Solna, Karolinska University Hospital and Karolinska Institutet, Sweden (J.F.L.)

**Keywords:** autoimmunity, celiac disease, cohort studies, heart, inflammation, dilatation

## Abstract

**Background:**

Dilated cardiomyopathy (DCM) is a rare disease of largely unknown origin. Previous studies have suggested an increased prevalence of celiac disease (CD) in patients with DCM. These studies, however, were based on a maximum of 5 patients with both CD and DCM. In the present large Swedish population-based cohort study, we examined the risk of idiopathic DCM in patients with CD determined by small-intestinal histopathology.

**Methods and Results:**

From 2006 to 2008, we collected duodenal/jejunal biopsy data on CD (equal to villous atrophy, Marsh stage 3, n=29 071 unique individuals) from (all) 28 pathology departments in Sweden. These individuals were compared with 144 429 reference individuals matched for age, sex, calendar year, and county. Data on DCM were obtained through the National Patient Register and confirmed by patient charts and echocardiography data. During follow-up, 17 patients with CD and 52 reference individuals developed idiopathic DCM. Thus, patients with CD were at an increased risk of idiopathic DCM (hazard ratio, 1.73; 95% confidence interval, 1.00 to 3.00), although the risk estimate failed to attain statistical significance (*P*=0.052).

**Conclusion:**

This nationwide study found a moderately but not statistically significantly increased risk of idiopathic DCM in patients with biopsy-verified CD. **(*J Am Heart Assoc*. 2012;1:e001594 doi: 10.1161/JAHA.112.001594.)**

## Introduction

Celiac disease (CD) affects approximately 1% of the European and North American population.^1^ On gluten exposure, these individuals develop an immune-mediated response in the small intestine that is characterized by inflammation, crypt hyperplasia, and villous atrophy. CD has been associated with an increased risk of not only ischemic heart disease^2,3^ and atrial fibrillation^4^ but also cardiovascular death,^5^ which suggests that CD is systemic, affecting cardiovascular morbidity.

Dilated cardiomyopathy (DCM) is defined by the presence of left ventricular dilatation and left ventricular systolic dysfunction in the absence of abnormal loading conditions (hypertension, valvular disease) or coronary artery disease sufficient to cause global systolic impairment.^6^ Familial forms with autosomal dominant inheritance and associations to X-linked muscular diseases and metabolic disorders exist,^6^ but otherwise the cause is largely unknown. Acquired forms of DCM may be due to nutritional deficiencies, endocrine disorders, and administration of cardiotoxic drugs, but also excessive alcohol intake, pregnancy, or (chronic) cardiac infections.^6^ Observations suggest that immunologic factors might be involved in the pathophysiology of DCM, which is also supported by a positive response to immunologic treatment in preliminary studies.^7–9^ The condition is serious, and in a Swedish study the 5-year survival rate was as low as 58%.^10^ A later study from Japan with modern treatment of DCM showed a 5-year survival rate of 80.9%.^11^

Most research on CD and DCM^12–14^ has examined the prevalence of CD in patients with DCM. To our knowledge, only 2 studies have examined the risk of DCM in CD,^15,16^ but these studies were restricted to information on CD and DCM from inpatient registers. Reliance on inpatient data on CD may overestimate relative risks, in that patients with CD admitted to hospital often have a more severe CD than the average patient with CD. In recent years, most patients with CD have been diagnosed as outpatients. Although most earlier studies^12–16^ found a positive association between CD and DCM, a recent Italian study found no increased risk of DCM in CD,^17^ and notably, no study so far has been based on >4 patients with both CD and DCM, resulting in very wide 95% confidence intervals (CIs).

In the present population-based study, we examined the risk of idiopathic DCM in patients with biopsy-verified CD. We hypothesized that DCM would be more common in patients with CD than in reference individuals.

## Methods

We linked nationwide histopathology data on CD with inpatient and outpatient data on DCM. We then confirmed the DCM diagnosis through patient chart reviews and echocardiography data before estimating the risk of idiopathic DCM in patients with CD. All charts were reviewed by L.E. A second reviewer (B.A.) evaluated 35 patient charts for which doubts existed about the DCM diagnosis.

### Collection of Biopsy Data

From October 2006 to February 2008, we collected data from biopsy reports at Sweden's 28 regional pathology departments.^18^ The biopsies had been performed since 1969, but because this study considered only computerized data, most of the biopsies were performed after 1990. CD was defined as having a Systematized Nomenclature of Medicine (SNOMED) classification code equal to villous atrophy in a biopsy from the small intestine^18^ (Marsh stage 3; for a detailed list of relevant morphology codes, see the Appendix). Small-intestinal biopsy is routine in clinical practice in Sweden before CD diagnosis; in fact, 96% to 100% of all gastroenterologists and pediatricians perform biopsy before CD diagnosis,^18^ and 95% of individuals with villous atrophy have CD.^18^ We did not request positive CD serology for a diagnosis of CD, but of patients with available data, 88% were serology positive at diagnosis.^18^

**Figure 1.**
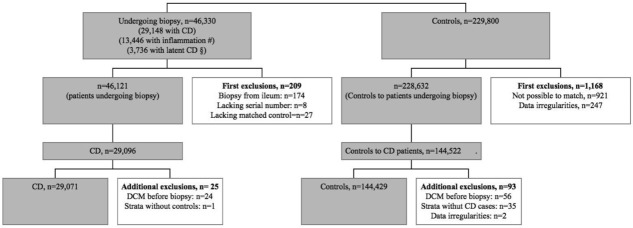
Flowchart of study participants. *Thirty-five reference individuals were excluded because their matched individual undergoing biopsy had been excluded (and all analyses were matched on strata; see Methods).

After the exclusion of duplicates and biopsies with data irregularities (eg, biopsy performed before birth or after death of the individual), data were available from 29 096 unique individuals with CD. These records were then sent for matching by the government agency Statistics Sweden. Additional exclusions were applied because of data irregularities or prior DCM (Figure 1). We used data from the National Patient Register (NPR),^19^ not patient chart data, to identify individuals with a prior diagnosis of DCM. Initially, we identified 199 patients with a DCM diagnosis in the NPR^19^ occurring after study entry. We then obtained special permission from the ethics review board and the Swedish National Board of Health and Welfare to access patient charts to confirm or reject the DCM diagnosis.

### Reference Individuals

For each individual with CD, Statistics Sweden identified up to 5 reference individuals matched for age, sex, county, and calendar year from the Total Population Register.^20^ For example, a woman diagnosed with CD at the age of 68 years in 1995 was compared with up to 5 reference women who were 68 years of age in 1995. The Total Population Register includes information on the Personal Identity Number, area of residence, sex, age, civil status, and date of emigration for all Swedish residents. The register was computerized in 1967, and data are collected and updated continuously by the local tax offices. Therefore, it contains all Swedish citizens. Reference individuals were sampled from all Swedish residents for whom the 28 regional pathology registers had not indicated prior duodenal/jejunal biopsy.

### Outcome Measure

We identified 199 individuals with a relevant DCM code in the Swedish NPR,^19^ which consisted of both inpatient and hospital-based outpatient data (codes: *International Classification of Diseases, 9th ed* [ICD-9]: 42.5E; ICD-10: I42.0). We obtained patient charts from 166 (83.4%) of the 199 individuals for evaluation.

Patients were identified as having “true idiopathic DCM” if they had a relevant ICD code and (1) positive echocardiographic examination (ejection fraction <40% and left ventricular diastolic diameter >32 mm/m^2^ of the body volume), (2) a coronary angiography without >50% stenosis, and (3) no record of other known causes of heart failure and cardiomyopathy, such as ischemic heart disease, valvular failure, or severe hypertension (see exclusion criteria in the Appendix). Patients were defined as having “probable DCM” if they had a relevant ICD code plus a positive echocardiography but other potential contributing factors could not be completely ruled out or coronary angiography had not been performed. Our main outcome measure consisted of “true DCM” or “probable DCM.” Sixty-nine study participants fulfilled our criteria for true or probable DCM: 17 patients with CD, of whom 8 had true DCM, and 52 reference individuals, of whom 26 had true DCM.

### Other Covariates

Earlier versions of the Swedish ICD (versions 7 through 9) did not distinguish between type 1 and type 2 diabetes. We therefore defined type 1 diabetes as having a relevant ICD code (ICD-8: 250; ICD-9: 250; ICD-10: E10) recorded before the age of 30 years at hospital admission in the Swedish NPR. Autoimmune thyroid disease and rheumatoid arthritis were defined according to relevant ICD codes, as listed in the Appendix. When adjusting for education, we used 7 a priori–defined educational categories determined by Statistics Sweden.

### Statistical Analyses

We examined the hazard ratio (HR) for idiopathic DCM with stratified Cox regression: Each individual with CD was matched with up to 5 reference individuals for age at first biopsy (and corresponding age in reference individuals), calendar year, sex, and county. Each index individual with CD was then compared only with his or her matched reference individuals, and only after that was a summary HR estimated. The consequence of this statistical approach is that the influence of the matching variables on the HR for DCM was eliminated.

Follow-up started on the date of first biopsy and the corresponding date in matched reference individuals. Follow-up ended with a confirmed diagnosis of idiopathic DCM, death, or emigration, or on December 31, 2008, whichever occurred first. In reference individuals, follow-up ended if the individual had a small-intestinal biopsy.

In a priori analyses, we examined the risk of idiopathic DCM according to follow-up (<1, 1 to 4.99, and ≥5 years), sex, age (0 to 19, 20 to 39, 40 to 59, and ≥60 years at first biopsy), and calendar period of first biopsy (before or during 1989, 1990 to 1999, and during or after 2000). We also examined the HR for all patients with an ICD code of DCM (independently of the result of the patient chart validation) and for patients who were regarded as having “true DCM” after chart validation.

In a separate analysis, we adjusted for education as well as type 1 diabetes, thyroid disease, and rheumatoid arthritis.

Statistical significance was defined as 95% CI for risk estimates not including 1.0. We used SPSS version 18.0 (SPSS Inc, Chicago, IL) for all analyses.

### Post Hoc Analyses

We also examined the risk of having a prior diagnosis of DCM using conditional logistic regression to calculate odds ratios. The case–control analysis was based on 29 096 individuals with CD and 144 522 reference individuals matched for sex, age, county, and calendar year. We did not have access to patient charts in patients with a DCM diagnosis before CD diagnosis, and thus we were able to examine only the association between CD and earlier DCM of any kind.

### Ethics

This project (2006/633–31/4) was approved by the Research Ethics Committee of the Karolinska Institute, Sweden, on June 14, 2006. None of the funders had any role in the design and conduct of the study; collection, management, analysis, and interpretation of the data; or preparation, review, and approval of the manuscript.

## Results

### Validation of DCM Through Patient Charts

Our validation of medical charts of the initial 166 patients with a register-based diagnosis of DCM revealed that valvular failure and other systemic diseases were more common in patients with concomitant CD (Appendix). Reference individuals with DCM more often had hypertension, excess alcohol intake, and arrhythmia (Appendix). Nevertheless, the difference between patients with CD and reference individuals was statistically significant only for excess alcohol intake.

### Background Data of Patients With Verified DCM

The majority of study participants were female (Table 1). The median age at first recorded DCM diagnosis was 62 years for patients with CD and 58 years for reference individuals. Seventy-six percent of the patients with CD and DCM and 50% of the reference individuals with DCM were men. This difference was not statistically significant (Table 2). Seventy-six percent of patients with CD and DCM and 79% of reference individuals with DCM were hospitalized on some occasion because of DCM. Two patients with CD and 8 controls improved markedly in symptoms and ejection fraction after medical treatment had begun.

**Table 1:** Characteristics of Study Participants

		Matched Reference
	Patients With CD	Individuals
Total number of participants	29 071	144 429

Age, y		

At study entry, median (range)	30 (0–95)	30 (0–95)

0–19, n (%)	11 799 (40.6)	58 850 (40.7)

20–39, n (%)	5309 (18.3)	26 376 (18.3)

40–59, n (%)	6469 (22.3)	32 217 (22.3)

≥60, n (%)	5494 (18.9)	26 986 (18.7)

Follow-up,* y		

Median (range)	9.1 (0–39.6)	9.0 (0–39.5)

Mean±SD	10.4±6.4	10.3±6.4

Sex, n (%)		

Female	18 001 (61.9)	89 526 (61.9)

Male	11 070 (38.1)	54 903 (38.1)

Calendar year, n (%)		

Before or during 1989	4105 (14.1)	20 375 (14.1)

1990–1999	12 054 (41.5)	59 841 (41.4)

During or after 2000	12 912 (44.4)	64 213 (44.5)

Entry year, median (range)	1998 (1969–2008)	1998 (1969–2008)

Type 1 diabetes,† n (%)	922 (3.2)	536 (0.4)

Autoimmune thyroid disease, n (%)	204 (0.7)	324 (0.2)

Age at DCM diagnosis, y, median (range)	58 (17–82)	64 (2–87)

Age from study entry to DCM diagnosis, y, median (range)	6 (0–23)	7 (0–27)

* Follow-up until diagnosis of DCM, death, emigration, or December 31, 2008. In reference individuals follow-up can also end through small-intestinal biopsy.

† Type 1 diabetes was defined according to age at first diagnosis with diabetes mellitus (<30 y).

**Table 2:** Characteristics of Patients With DCM

	Patients With CD (n=17)	Reference Individuals (n=52)	*P*
Sex, n (%)			0.06

Men	13 (76)	26 (50)	

Women	4 (24)	26 (50)	

Age at CD diagnosis, y, n (%)			0.07

0–19	1 (6)	4 (7)	

20–39	1 (6)	5 (10)	

40–59	10 (59)	40 (77)	

≥60	5 (29)	3 (6)	

Age at DCM diagnosis, y, median (range)	62 (40–78)	58 (2–74)	

No information on coronary angiography, n (%)	9 (53)	17 (33)	0.14

Hypertension, n (%)	1 (6)	7 (13)	0.39

History of diabetes, n (%)	3 (18)	10 (19)	0.88

Excess alcohol intake, n (%)	0 (0)	5 (10)	0.18

Primary valvular failure, n (%)	2 (12)	1 (2)	0.08

History of other systemic disease, n (%)	3 (18)	8 (15)	0.82

Any type of arrhythmia, n (%)	3 (18)	5 (10)	0.37

Record of DCM heredity, n (%)	0 (0)	0 (0)	

Onset during pregnancy, n (%)	0 (0)	1 (2)	

Laboratory data			

Hemoglobin, mean±SD	139±14	140±12	0.81

C-reactive protein, median (range)	5 (5–40)	3 (2–3)	*

* Mann-Whitney *U* test showed no significant difference.

### Main Analysis

The HR for chart-verified DCM in CD was 1.73 (95% CI, 1.00 to 3.00; *P*=0.052). Adjustment for diabetes, rheumatoid arthritis, country of birth, or level of education did not influence the risk estimates, but with adjustment for autoimmune thyroid disease, the HR reached statistical significance (HR, 1.76; 95% CI, 1.01 to 3.05).

The association between CD and DCM was seen in the first 5 years after diagnosis (Table 3). Stratifying the analysis according to sex revealed a lower HR for DCM in women with CD (HR, 0.89; 95% CI, 0.31 to 2.56) than in men with CD (HR, 2.47; 95% CI, 1.26 to 4.83) (Table 4). The excess risk per 100 000 person-years at risk was 6.6 (absolute risk 11.6) for men and −0.7 (absolute risk 2.1) for women, but this may have been a chance finding because *P* for interaction between CD and sex was not <0.05 (*P*=0.06). The HR was elevated during the first 5 years after CD diagnosis but was neutral >5 years after diagnosis (Table 2). Use of age instead of time since biopsy as the time scale did not change our risk estimate (HR, 1.76; 95% CI, 1.01 to 3.05). With restriction of our outcome to “true DCM,” the HR was 1.26 (95% CI, 0.48 to 3.35).

**Table 3:** DCM According to Follow-Up

Subgroup/Exposure	Events	HR (95% CI)	*P*	PYAR	Absolute Risk / 100 000 PYAR	Excess Risk / 100 000 PYAR
All						

Reference	52	1 (reference)		1 501 573	3	

CD	17	1.73 (1.00–3.00)	0.052	298 830	6	3

Year <1						

Reference	4	1 (reference)		143 730	3	

CD	3	4.06 (0.91–18.19)	0.067	28 745	10	7

Years 1–4.99						

Reference	11	1 (reference)		516 230	2	

CD	7	3.36 (1.29–8.71)	0.013	102 906	7	5

Years ≥5						

Reference	37	1 (reference)		841 613	4	

CD	7	0.99 (0.44–2.24)	0.982	167 179	4	0

PYAR indicates person-years at risk.

Reference is general population comparator cohort.

**Table 4:** Subgroup Analyses in Relation to Risk of DCM

Subgroup	Observed Events	HR (95% CI)	*P*
Sex			

Male	13	2.37 (1.26–4.83)	0.008

Female	4	0.89 (0.31–2.56)	0.829

Age, y			

<20	1	1.34 (0.15–11.97)	0.769

20–39	1	0.96 (0.11–8.24)	0.971

40–59	10	1.32 (0.66–2.65)	0.439

≥60	5	9.50 (2.25–40.17)	0.002

Calendar period			

Before or during 1989	4	1.87 (0.59–5.98)	0.290

1990–1999	9	1.49 (0.71–3.15)	0.292

During or after 2000	4	2.37 (0.74–7.62)	0.149

PYAR indicates person-years at risk.

Reference is general population comparator cohort.

When we used any DCM (n=199, identified through the NPR regardless of patient chart information) as our outcome measure, the HR decreased to 1.31 (95% CI, 0.91 to 1.88), probably because of lower specificity for any DCM, in that positive events in this analysis had not been confirmed by chart validation or echocardiography data. In addition, many of the patients with any DCM may not have had idiopathic DCM but rather other types of cardiomyopathy or DCM, explained by factors such as ischemic heart disease or valvular failure.

### Risk of DCM Before CD

A prior diagnosis of DCM in the Swedish NPR was associated with a later diagnosis of CD (HR, 1.99; 95% CI, 1.27 to 3.12). There were 24 events of prior DCM in the CD group and 56 events in the reference individual group.

## Discussion

This is the first population-based cohort study of DCM in CD in which the DCM diagnosis has been confirmed against patient chart and echocardiography data. We found a 73% increased risk of DCM in CD, and the risk was highest in the first year after CD diagnosis.

Several studies have reported an increased prevalence of CD in patients with DCM. However, the number of patients has been relatively small, ranging from 1 to 4 patients with both CD and DCM diagnoses in each study.^12,14,15^ The present study identified 17 patients with CD and patient chart–validated idiopathic DCM.

Our findings of an increased risk of DCM in CD are consistent with previous reports.^12,15^ Even though our risk estimate was of only borderline significance, it was consistently about 1.7 and reached statistical significance in several subanalyses (eg, when we adjusted for other autoimmune thyroid disease and when we used age instead of time since CD diagnosis as the time scale). We therefore suggest that CD is associated with an increased risk of DCM.^21^

The present study found a 4-fold increased risk of idiopathic DCM in the first year after CD diagnosis. The inflammation and systemic reactions of CD are most intense around the time of diagnosis, an event that could potentially trigger the onset of DCM. Still, we cannot rule out the possibilities that ascertainment bias contributed to the risk increase and that patients with newly diagnosed CD underwent investigations for multiple symptoms and signs leading up to a diagnosis of DCM.

Although CD is more common in women,^22^ 13 (76%) of 17 individuals with both CD and DCM were male. This sex distribution is consistent with earlier data on DCM,^23,24^ as well as data on patients with CD and DCM.^25^ Although the proportion of males in reference individuals with DCM in this cohort was lower (50%), it did not attain statistical significance.

The main strengths of our study include its large statistical power compared to earlier studies on CD and DCM, the population-based design, confirmation of the CD diagnosis through biopsy, and confirmation of the DCM diagnosis against patient charts and echocardiography. Such validation increases diagnostic accuracy. The HR for DCM in CD was higher than for some other heart outcomes that we have investigated previously but in which immunologic mechanisms may be of lesser importance (ischemic heart disease: HR, 1.19 ^3^; atrial fibrillation: HR, 1.34 ^4^). The inclusion of all Swedish patients with computerized records of biopsy-verified CD, in combination with extensive chart validations to confirm the DCM diagnosis, indicates that our study has high external validity.

This study also has some limitations. We had no information on body mass index, but so far no strong association between obesity and DCM has been established; the only study we know of found an HR of 1.1.^26^ We also lacked information on smoking, despite the fact that DCM has been associated with passive^27^ and likely also with active smoking.^28^ Although smoking and possibly high body mass index are potential risk factors for DCM, most evidence suggests they are negatively associated with CD.^29,30^ Hence, the lack of data on smoking and body mass index cannot explain the positive association between CD and DCM found in our study. We also had no individual-based information about dietary adherence, and we cannot rule out the possibility that DCM mostly occurred in patients with CD with poor dietary adherence. In a subset of individuals with CD, 17% had signs of poor dietary adherence.^18^

The positive association between CD and DCM may have several explanations, including nutritional deficiencies. Iron deficiency anemia is often seen in cardiomyopathy, and iron deficiency anemia is common in patients with CD.^13^ Carnitine deficiency, common in patients with CD,^31^ also has been shown to be more severe in patients with CD and DCM than in patients with isolated DCM.^32^

The most plausible explanation for the link between CD and DCM is that both conditions might be mediated through inflammation and autoimmune mechanisms. Chronic inflammation is common in CD, both before and after diagnosis,^33^ and may influence myocardial function. Although our study found no statistically significant difference in C-reactive proteinv levels between patients with CD and reference individuals, inflammation in CD may not be fully represented by C-reactive protein in that research has shown mainly increased levels of interleukin-4, interleukin-6, interleukin-10, and tumor necrosis factor-α, as well as other cytokines, in patients with CD.^34^ We did not have access to data on cytokine levels. Interleukin-10 also has been shown to be increased in patients with DCM.^7^

In conclusion, we found a statistically nonsignificant positive association between CD and DCM, both before and after diagnosis, suggesting that the 2 diseases may share etiology or that inflammation in undiagnosed and diagnosed CD may trigger DCM.

## Supplementary material

Disclaimer: Supplementary materials have been peer-reviewed but not copyedited.
